# T‐Helper Type 1 Bias in Healthy People Is Associated With Cytomegalovirus Serology and Atherosclerosis: The Multi‐Ethnic Study of Atherosclerosis

**DOI:** 10.1161/JAHA.113.000117

**Published:** 2013-06-21

**Authors:** Russell P. Tracy, Margaret F. Doyle, Nels C. Olson, Sally A. Huber, Nancy S. Jenny, Reem Sallam, Bruce M. Psaty, Richard A. Kronmal

**Affiliations:** 1 Department of Pathology, University of Vermont, Burlington, VT (R.P.T., M.F.D., N.C.O., S.A.H., N.S.J., R.S.); 2 Department of Biochemistry, University of Vermont, Burlington, VT (R.P.T.); 3 Departments of Medicine, Epidemiology and Health Sciences, University of Washington, Seattle, WA (B.M.P.); 4 Department of Biostatistics, University of Washington, Seattle, WA (R.A.K.); 5 Group Health Research Institute, Group Health Cooperative, Seattle, WA (B.M.P.); 6 College of Medicine, Clinical Chemistry Unit, Pathology Department, and Obesity Research Center, King Saud University, Riyadh, Kingdom of Saudi Arabia

**Keywords:** atherosclerosis, epidemiology, immunology, inflammation, T‐helper cell

## Abstract

**Background:**

Although T‐helper type 1 (Th1) cells are considered important in atherosclerosis, the relationships between Th1 and Th2 cells and atherosclerosis have not been examined in population‐based studies.

**Methods and Results:**

We measured Th cells as a percentage of lymphocytes by flow cytometry using CD4 staining (%CD4) in 917 participants of the Multi‐Ethnic Study of Atherosclerosis. We also measured interferon gamma–positive and interleukin‐4‐positive CD4^+^ cells, representing Th1 and Th2 subpopulations (%Th1 and %Th2), respectively. We found that %CD4 was 1.5% lower per 10 years of age (*P*<0.0001). Whites had higher %CD4 and lower %Th1 and %Th2 values than other race/ethnic groups. Body mass index (BMI) and blood pressure were associated with %CD4, but no traditional cardiovascular disease (CVD) risk factors were associated with %Th1 or %Th2. In multivariable models, the major independent variable associated with %Th1 was cytomegalovirus (CMV) antibody titer, with minor contributions from age, sex, seasonality, and interleukin‐6. In models with coronary artery calcification level as the outcome, significant independent variables included age, sex, smoking status, and %Th1 (β=0.25; *P*≤0.01). Both %Th1 and %Th2 were associated with common carotid intimal media thickness (β=0.02 and −0.02, respectively; both *P*<0.05), as were age, sex, race/ethnicity, blood pressure, and BMI.

**Conclusions:**

Th1 bias is associated with subclinical atherosclerosis in a multiethnic population. The main Th1 correlate was CMV infectious burden. These findings are consistent with a role of Th1 cells in atherosclerosis and suggest the importance of prospective studies of T‐helper cell biasing in CVD.

## Introduction

Although the importance of inflammation in atherosclerosis and cardiovascular disease (CVD) is well accepted,^1^ the underlying mechanisms and pathways remain incompletely defined. In epidemiological research, systemic biomarkers of chronic low‐level inflammation, such as fibrinogen and C‐reactive protein (CRP), are associated with subclinical atherosclerosis and are independently associated with future clinical CVD events.^2^ As acute‐phase proteins, these biomarkers are generally nonspecific, although each also plays a role in a specific inflammatory pathway—coagulation and innate immunity, respectively.

As recently reviewed by Hansson et al,^3–4^ aspects of the adaptive immune system also play important roles in atherosclerosis. In particular, T‐helper type 1 (Th1) cells provide important stimulation to macrophage and foam cells in mouse models of atherosclerosis via several mechanisms, including production of interferon gamma (IFN‐γ).^5^ Th1 bias drives increased atherosclerosis in mice,^6^ whereas deficiency in IFN‐γ or the Th1‐differentiating transcription factor T‐bet results in reduced lesion development.^7–9^ In relatively small clinical studies, subjects with coronary syndromes and advanced atherosclerosis were found to have a bias toward Th1.^10–15^ Th1 cells are also critically important in chronic inflammatory disease states such as psoriasis, which may confer an independent risk of myocardial infarction (MI).^16^ To date, information regarding the relationships between Th1 cells and atherosclerosis have been limited to small‐scale clinical studies in individuals with advanced CVD.

Little is known about the role of the other major T‐helper cell, the Th2 cell, which produces, among other cytokines, interleukin‐4 (IL‐4). Th2 cells can function in anti‐inflammatory capacities by inhibiting Th1 development^17^ and by the IL‐4‐mediated suppression of proinflammatory cytokine release.^18^ Accordingly, Th2 cells have been suggested to be antiatherogenic.^6^ On the other hand, Th2 cells are proinflammatory in conditions such as asthma^19^ and have also been reported as proatherogenic.^20–21^ Collectively, the role of Th2 cells in atherosclerosis remains unclear.

Little information concerning adaptive immunity and T‐helper function is available from population‐based studies of individuals free of CVD. Therefore, we designed and implemented a study in the setting of the Multi‐Ethnic Study of Atherosclerosis (MESA) called MESA‐Inflammation. We report here the associations between Th1 and Th2 cells and measures of subclinical atherosclerosis in otherwise healthy individuals.

## Methods

### Study Populations

MESA is a longitudinal epidemiological study of 6814 asymptomatic men and women aged 45 to 84; details have been published^22^ and are available online at http://www.mesa-nhlbi.org. Briefly, participants include whites, African Americans, Hispanic Americans, and Asian Americans recruited at 6 field centers (Baltimore, MD; Chicago, IL; Los Angeles, CA; Minneapolis, MN; New York, NY; Winston‐Salem, NC) during the baseline exam (exam 1) in 2000–2002. Exclusion criteria included: clinical CVD defined as physician‐diagnosed MI, angina, stroke, transient ischemic attack, heart failure, or atrial fibrillation; use of nitroglycerin; or a major procedure related to the treatment of CVD. Subsequently, exams 2, 3, 4, and 5 occurred in 2002–2004, 2004–2005, 2005–2007, and 2010–2012.

As part of exam 1, we selected a random subset of ≈1000 participants for specialized biomarker testing. During exam 4 (2005–2007), 716 participants from this subset plus 201 replacements for those who had died or dropped out were selected for MESA‐Inflammation. Samples were collected at all 6 field centers and sent overnight in specialized containers to the Laboratory for Clinical Biochemistry Research (LCBR) at the University of Vermont. Both the parent MESA study and MESA‐Inflammation received institutional review board approval, and informed consent was obtained.

### CVD Risk Factors, and Biomarkers

Standardized questionnaires were administered at the baseline exam in 2000–2002 and during each subsequent exam for demographics, medical history, medications, and lifestyle characteristics, anthropomorphic variables, diabetes status, smoking status, blood pressure, and so forth. At each exam fasting blood was drawn, and serum and plasma were prepared and shipped to the MESA Core Laboratory at the University of Vermont. Smoking was defined as never, former (no cigarettes within the past 30 days), or current. Impaired fasting glucose and type 2 diabetes were classified by the 2003 American Diabetes Association (ADA) criteria: fasting glucose between 100 and 125 and ≥126 mg/dL, respectively.^23^ Hypertension was classified according to JNC VI.^24^ Data from exam 4 were used except for variables such as specialized biomarkers only measured at baseline, including soluble intracellular adhesion molucule–1 (sICAM‐1; Parameter Human sICAM‐1 ELISA; R&D Systems, Minneapolis, MN; analytical coefficient of variation [CV], 5%), soluble interleukin‐2 receptor α (sIL‐2rα; Quantikine Human IL‐2 sRα ultra‐sensitive ELISA; R&D Systems, Minneapolis, MN; CVs ranged from 4.6% to 7.2%), and high‐sensitivity C‐reactive protein, interleukin‐6 (IL‐6), and infectious serologies, which were measured as described previously.^25–26^

### Laboratory Measurements

Lipid and glucose measurements from exam 4 were conducted at the Collaborative Studies Clinical Laboratory at the University of Minnesota Medical Center, Fairview (Minneapolis, MN). Serum glucose was measured on a Vitro analyzer (Johnson & Johnson Clinical Diagnostics, Inc, Rochester, NY); CV 1.1%. Lipid measurements were performed on EDTA plasma samples collected following an overnight fast. Total cholesterol and triglycerides were measured on a Roche Modular P Chemistry Analyzer (Roche Diagnostics, Indianapolis, IN), with CVs of 1.6% and 4.0%, respectively. HDL‐cholesterol was measured using the cholesterol oxidase method (Roche Diagnostics) following precipitation of non‐HDL‐cholesterol with magnesium/dextran; CV 2.9%. LDL‐cholesterol was calculated using the formula of Friedwald et al^27^ in plasma specimens having a triglyceride value <400 mg/dL.

### Subclinical Atherosclerosis Measures

Scanning centers assessed coronary artery calcification (CAC) by chest computed tomography using either a cardiac‐gated electron‐beam computed tomography scanner or a multidetector computed tomography system. Certified technologists scanned all participants twice with phantoms of known physical calcium concentration. A radiologist or cardiologist read all computed tomography scans at a central reading center (Los Angeles Biomedical Research Institute at Harbor‐UCLA in Torrance, CA). Carr et al^28^ have reported the details of the MESA computed tomography scanning and interpretation methods.

Trained technicians in each field center performed B‐mode ultrasonography of the right and left near and far walls of the internal carotid and common carotid arteries, using the Logiq 700 ultrasound device (General Electric Medical Systems, Waukesha, WI).^29^ An ultrasound reading center (Department of Radiology, New England Medical Center, Boston, MA) measured maximal intimal media thickness (IMT) of the internal carotid (IC) and common carotid (CC) sites as the mean of the maximum IMT of the near and far walls of the right and left sides.

CAC measurements were made on the full cohort in exam 1 and again in exams 2 and 3 (half the cohort in each exam) and in exam 4 (about one quarter of the cohort). The CAC data from the exam closest in time to exam 4 was used for these analyses (419 from exam 4, 426 from exam 3, 65 from exam 2, and 7 from exam 1). The common and internal carotid IMTs were measured at baseline and used for this study.

### Flow‐Cytometric Analyses of T‐Cell Phenotypes

#### Protocol development

Once cell populations were decided on, samples were tested for overnight stability. A loss in CD4^+^ cells in certain local volunteers on overnight incubation led us to examine blood‐draw tube types. Testing cell levels in blood drawn into heparin, EDTA, citrate, and acid‐citrate‐dextrose tubes that were tested immediately or allowed to sit overnight demonstrated that heparin was the best choice for reproducibility, particularly in those volunteers whose CD4^+^ cell levels appeared to drop overnight when compared with freshly measured cell levels. In addition, we tested different overnight storage temperatures and found that room temperature was most stable, whereas temperature extremes (4°C and 37°C) showed extensive hemolysis in the plasma, indicating cell damage. Testing was performed to confirm that the shipping boxes and ambient shipping packs were stable to temperature extremes as well as potential movement of packages such as seen with overnight shipping. These results were similar to results published by Nicholson et al^30^ in their study on stability of blood samples from HIV‐positive and ‐negative individuals, where they reported that heparin‐anticoagulated blood was more stable over time than EDTA and that ambient temperatures were most stable for cellular studies.

**Figure 1.**
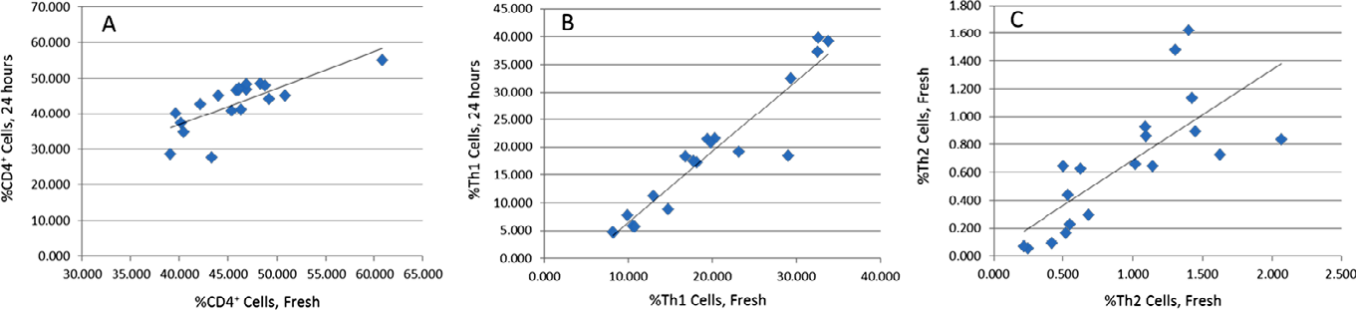
Linear regression analyses of cellular measurements in fresh whole blood versus 24‐hour postdraw samples. The *x* axis represents values from freshly processed whole blood, and the *y* axis represents values from 24‐hour postdraw processing of whole blood. A, %CD4^+^ cells from peripheral blood mononuclear cells (PBMCs); B, %Th1 cells; C, log %Th2 cells.

Once a method was developed, we measured our cell populations in freshly drawn versus 24‐hour postdraw samples subjected to standard shipping procedures in a group of 13 to 20 local, apparently healthy volunteers (Figure 1). Regression slopes comparing fresh versus 24‐hour postdraw cell measurements were 1.04 for CD4^+^ cells, 1.28 for Th1 cells, and 0.55 for Th2 cells, and their respective Pearson correlation coefficients were 0.75, 0.95, and 0.74, indicating that results were similar between the 2 samples.

Because of the large number of samples that are analyzed in population‐based studies, we examined the ability to fix the cells in paraformaldehyde for extended periods and found that samples could be fixed, stored for at least a week, and analyzed by flow cytometry in batches.

#### Finalized protocol

Heparinized blood (10 mL sodium heparin; BD Biosciences, San Jose, CA) was drawn at the MESA field centers, packaged in Saf‐T‐Pak Styrofoam shipping containers (Glen Burnie, MD) with 4 gel packs that maintained a temperature of 15°C to 30°C, and shipped via overnight courier to the LCBR at the University of Vermont. Tubes were placed on a shaker for 10 minutes before the start of the protocol. Heparinized blood was centrifuged at 1500 RCF for 15 minutes at room temperature. The cells were resuspended in 35 mL of fully supplemented RPMI‐1640 ([fsRPMI; Invitrogen, Carlsbad, CA] containing 10% fetal bovine serum [Mediatech, Inc., Manassas, VA], 2 mmol/L l‐glutamine [Mediatech, Inc.], and 100 IU/mL penicillin/100 ug/mL streptomycin [Mediatech, Inc.]). Histopaque (Sigma‐Aldrich, St. Louis, MO) was layered under the cell suspension and then centrifuged as above. The peripheral blood mononuclear cell (PBMC) layer was removed and washed with fsRPMI, and the cell pellet was resuspended in fsRPMI. Cells were counted using a Beckman Coulter Z‐2 Counter (Beckman Coulter, Danvers, MA) and adjusted to a concentration of 1x10^7^ cells/mL.

All reagents and antibodies were from Sigma‐Aldrich (St. Louis, MO) unless otherwise noted. Isolated PBMCs were stimulated with 40 ng/mL phorbol 12‐myristate 13‐acetate (PMA), 1 μg/mL ionomycin, and 10 μg/mL Brefeldin A for 3 hours at 37°C in a humidified atmosphere containing 5% CO_2_. Following stimulation, PBMCs were washed with phosphate‐buffered saline (PBS) containing 1% bovine serum albumin (BSA) and 10 μg/mL Brefeldin A. As negative controls, unstimulated PBMCs that underwent the same protocol without the addition of activators showed no cytokine staining.

The stimulated PBMCs were incubated with Fc Block (BD Biosciences) to block nonspecific antibody‐binding sites, then labeled with R‐phycoerythrin‐cyanine dye Cy5 (PE‐Cy5)‐conjugated anti‐CD4 (BD Biosciences) to surface‐label the CD4‐positive (%CD4^+^) cells or with PE‐Cy5‐conjugated mouse IgG1 (BD Biosciences) as an isotype control. After washing with PBS/1% BSA/Brefeldin A, cells were fixed using 2% paraformaldehyde (Alfa‐Aesar, Ward Hill, MA) for 15 minutes. Cells were washed and then permeabilized using 0.1% saponin in PBS/1% BSA. Cells were then washed and incubated with fluorescein isothiocyanate (FITC)–conjugated anti‐IFN‐γ and PE‐conjugated anti‐IL‐4 for 20 minutes in the dark, according to the manufacturers’ instructions. FITC‐conjugated mouse IgG2b κ and PE‐conjugated mouse IgG1 κ were analyzed as isotype‐matched controls. Cells were fixed in 2% paraformaldehyde and were maintained at 4°C protected from light until evaluated.

Cells were analyzed by flow cytometry using an LSR II flow cytometer (BD Biosciences) with a single excitation wavelength (488 nm) and band filters for PE‐Cy5 (670/14 nm), FITC (530/30 nm), and PE (575/26 nm). Single color controls were used to set machine compensation, and background staining was determined using the respective negative isotype control. Data was analyzed using Winlist 6.0 (Verity Software House, Topsham, ME). Lymphocytes (30 000 events) were gated based on their forward and side scatter. T‐helper lymphocytes were gated by positive surface staining for CD4 and were expressed as a percentage of gated lymphocytes; Th1 cells (CD4^+^IFN‐γ^+^) and Th2 cells (CD4^+^IL‐4^+^) were expressed as a percentage of CD4^+^ cells.

#### Quality assurance

In an effort to ensure there was no drift in our assay protocol, we enlisted 14 local volunteers who donated samples before the commencement of the study and periodically throughout the study. This also allowed us to assess biovariability. Using nested analysis of variance, as described by Sakkinen et al^31^ and originally proposed by Fraser,^32–33^ we were able to determine 3 coefficients of variation: analytical (CVa), within subject (CVi), and between subjects (CVg). In cases in which analysis was done only in singletons, no CVa was available. From these data, an index of individuality (II), which is the ratio of the CVi to CVg, was determined. The II is a measure of the ability to detect changes in an individual compared with the total population. In general, assays with an II of <1 have proven useful in population studies. These biovariability data are summarized in Table 1. The results indicated that these analytes are reasonably stable phenotypes. In fact, the Th1 values (II=0.43) are very similar to cholesterol (II=0.44), a widely accepted biomarker.^31^

**Table 1.** Biovariability Measures for Cellular Phenotypes

Measure	Mean (SD)	% CVa	% CVi	% CVg	II	CVg/CV_total_
%CD4^+^ PBMCs	43.7 (9.9)	3.65	21.10	16.84	1.25	0.40
%CD4^+^ WB	38.4 (8.0)	—	15.87	12.63	1.26	0.44
%Th1	16.5 (10.2)	3.73	26.25	60.60	0.43	0.67
Log %Th2	0.23 (0.15)	14.05	50.65	46.59	1.09	0.42

Data are means (SDs). CVa indicates analytic coefficient of variation; CVi, intraperson coefficient of variation; CVg, interperson coefficient of variation; II, index of individuality (CVi/CVg); CV_total_, CVa+CVi+CVg; %CD4^+^ PBMCs, CD4^+^ cells measured from preparations of peripheral blood mononuclear cells; %CD4^+^ WB, CD4^+^ cells measured in whole blood as a comparison.

In an effort to minimize errors from this protocol, we established a checklist for quality assurance. Using these procedures over a 2‐year period, receiving samples 4 days per week, we lost only 20 overnight samples to late arrival, generally because of weather affecting delivery. A few data points were lost to a backordered reagent, and a corrupt flow‐cytometry data file caused the loss of ≈2% of the data. Together, the assay development and quality assurance plan implemented as part of the MESA‐Inflammation Study allowed us to generate high‐quality cellular data on 917 participants.

### Statistical Analyses

Descriptive displays of means or Pearson correlations were used to describe associations between cell phenotypes and other variables. Linear regression models were constructed with %Th1, %Th2, and %CD4 cell indices as outcomes using backward elimination (*P*>0.05 for elimination). After forcing age, sex, and race/ethnicity into the models, body mass index (BMI), season, cytomegalovirus (CMV) antibody titers, *H. pylori* index values, hepatitis A index values, HSV antibody titers, *C. pneumoniae* fluorescence units, CRP, white blood cell count, IL‐6, smoking status (never, former, current), total cholesterol, HDL‐cholesterol, systolic blood pressure, diastolic blood pressure, use of hypertension medication, diabetes status, and education in 3 categories were allowed to enter the model. These same candidate variables were used in analyses of T‐cell indices with subclinical disease. In these analyses only 716 cases were included because CMV antibody titers were only measured in the original sample of 1000 participants.

A similar approach was used to model the association of %Th1, %Th2, and %CD4 with the subclinical disease measures. All the models started with all 3 cell‐based indices, which were eliminated if nonsignificant. Because the significant independent variables associated with the presence of CAC (CAC=0 versus CAC>0) were different from those associated with the amount of CAC in those with CAC present, we modeled presence of CAC separately from CAC amount. The former was modeled using relative risk regression and the latter with linear regression. For the linear regression models, robust regression was done to provide protection against the influence of influential outliers, which are potentially present for both CAC and the IMT measures; ln‐transformed Agatston scores were used in these analyses. Relative risk regression was used rather than logistic regression because the parameter of interest is relative risk rather than the odds ratio, and the odds ratio overestimates relative risk when the outcome is common (≈50% of the participants have CAC>0).

## Results

### T‐Cell Phenotypes in MESA

In the MESA‐Inflammation subgroup, 47% were treated for hypertension, 32% were treated for dyslipidemia, 14% were diabetic, and only 9% were current smokers (Table 2). The mean value of CRP at 3.8 mg/L is modestly high.

**Table 2.** Description of the MESA Sample Population Being Studied

Variable	MESA‐Inflammation
n	917
Age (y), mean (SD)	65.6 (9.9)
Male sex, n (%)	438 (48)
Ethnicity, n (%)	
White	398 (43)
African American	188 (21)
Hispanic American	235 (26)
Asian American	96 (10)
BMI (kg/m^2^), mean (SD)	28.7 (5.6)
Smoking status, n (%)	
Never	406 (45)
Former	421 (46)
Current	85 (9)
Diabetes status, n (%)	
Normal	570 (62)
Diabetes (takes medications)	132 (14)
Hypertension status, n (%)	
Normal	463 (53)
Hypertensive (takes medications)	418 (47)
Lipid status, mean (SD)	
LDL cholesterol, mg/dL	111 (34)
HDL cholesterol, mg/dL	52.4 (15.0)
Triglycerides, mg/dL	124 (72)
Inflammation status	
C‐reactive protein, mg/L (median, 25th, 75th)	1.98 (0.86, 4.43)
IL‐6, pg/mL (median, 25th, 75th)	1.15 (0.75, 1.79)
Soluble IL‐2 receptor α, ng/mL (median, 25th, 75th)	0.85 (0.68, 1.09)
Intercellular adhesion molecule‐1, pg/mL (mean, SD)	282 (82)
Atherosclerosis status	
Presence of coronary calcification, n (%)	541 (59)
CAC in positives, AU (median, 25th, 75th)	99.8 (24.3, 382.8)
IMT, common carotid, mm (mean, SD)	0.87 (0.19)
IMT, internal carotid, mm (mean, SD)	1.03 (0.55)
T‐lymphocyte indices, mean (SD)	
%CD4 (mean, SD)	42.1 (14.1)
%Th1 (median, 25th, 75th)	14.5 (9.8, 20.0)
%Th2 (median, 25th, 75th)	0.63 (0.35, 1.08)

This table includes those MESA‐Inflammation participants with ≥1 of the 3 cell‐based measurements: %CD4, %Th1, %Th2. The number of participants in individual cells may vary to a small degree. MESA indicates Multi‐Ethnic Study of Atherosclerosis; BMI, body mass index; LDL, low‐density lipoprotein; HDL, high‐density lipoprotein; IL, interleukin; CAC, coronary artery calcification; IMT, intimal media thickness.

**Figure 2.**
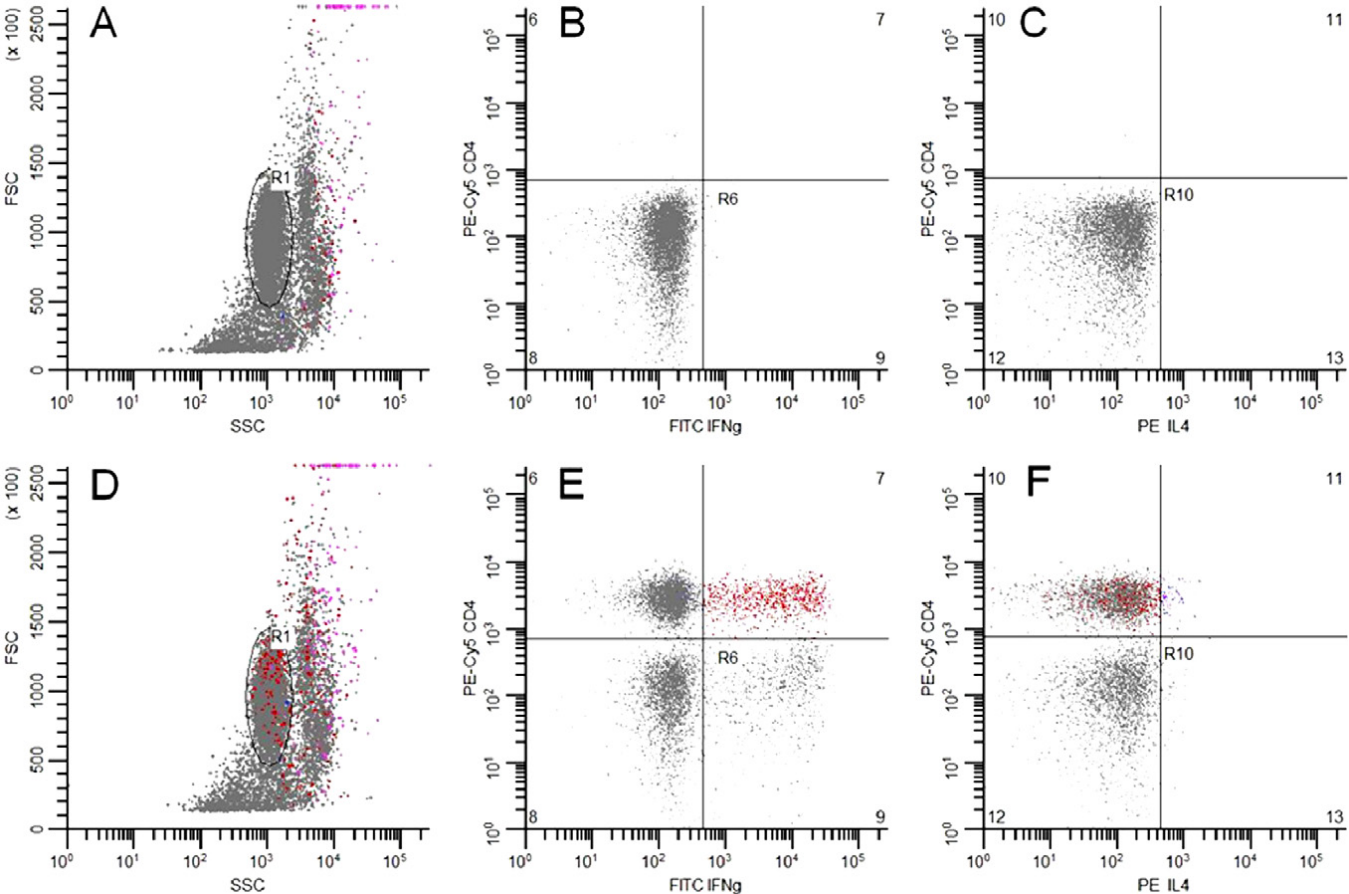
CD4, Th1, and Th2 flow‐cytometry gating strategies. The lymphocyte population is gated based on forward (*y* axis) and side (*x* axis) scatter plots using a fluorescently labeled sample (D) compared with an isotype control (A). Th1 cells are identified as those cells that are CD4^+^ (*y* axis) and IFN‐γ^+^ (*x* axis) (E) when compared with an isotype control (B). Th2 cells are identified as those cells that are CD4^+^ (*y* axis) and IL‐4^+^ (*x* axis) (F) compared with an isotype control (C). FSC indicates forward scatter; SSC, side scatter; FITC IFNg, fluorescein isothiocyanate‐conjugated anti‐IFN‐γ; PE IL4, R‐phycoerythrin‐conjugated anti‐IL‐4; IFN‐γ, interferon gamma.

Figure 2 illustrates a typical set of flow‐cytometric data for the MESA‐Inflammation study. A comparison of Figure 2E and 2F illustrates the relative ease and difficulty of estimating the %Th1 and %Th2 populations, respectively, because of both the number of cells being counted and the degree of separation between the positive‐ and negative‐staining cells.

**Figure 3.**
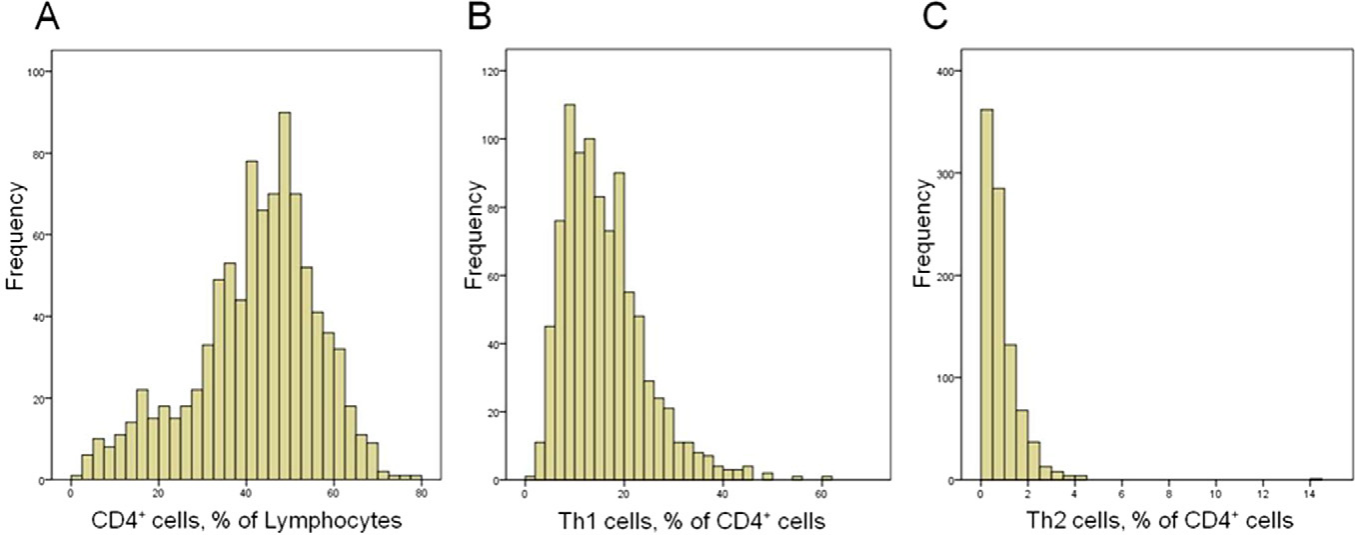
Distributions of cell phenotypes in Multi‐Ethnic Study of Atherosclerosis (MESA)–Inflammation. *y* Axis, count; *x* axis, value. A, %CD4; B, %Th1; C, %Th2.

Figure 3A through 3C shows distributions of %CD4, %Th1, and %Th2 cells. %Th1 and %Th2 were modestly skewed to the right; average values are given in Table 2.

### Correlates of %CD4, %Th1, and %Th2 Cell Phenotypes

Unadjusted associations of these phenotypes with CVD risk factors, biomarkers of inflammation, and infectious serologies are shown in Table 3. Older MESA participants had slightly lower %CD4 values (1.5%/10 years), and women had slightly higher %CD4 values; there was no association of age or sex with %Th1 or %Th2 values. Whites had markedly lower %Th1 and %Th2 values than the other ethnicities. Although BMI and blood pressure were associated with higher and lower %CD4 cells, respectively, no traditional CVD risk factor was associated with %Th1 or %Th2. There was some evidence for a weak association of systemic inflammation with cell values; the strongest was for IL‐6 and %Th1, with ≈0.1% higher %Th1 for each SD increase in IL‐6. We observed a significant difference in %Th2 by seasonality, a known correlate of immune function,^34^ with highest values in summer and lowest in winter; results were similar for %Th1 but not statistically significant.

**Table 3.** Unadjusted Associations of %CD4, %Th1, and %Th2 With Demographic and Other Variables

Variable	Mean (SD) or Correlation Coefficient					
	%CD4	*P* Value	%Th1	*P* Value	%Th2	*P* Value
Demographics						
Age	−0.15	≤0.0001	−0.03	NS	0.04	NS
Sex						
Women	41.5 (9.9)	≤0.0001	16.0 (8.5)	NS	0.85 (0.93)	NS
Men	38.2 (10.2)		15.9 (8.1)		0.84 (0.68)	
Race/Ethnicity						
White	42.4 (9.7)	≤0.0001	14.2 (7.4)	≤0.0001	0.71 (0.59)	≤0.0001
Asian American	35.0 (11.0)		16.9 (7.6)		1.04 (1.53)	
African American	40.2 (10.0)		17.1 (10.0)		0.98 (0.78)	
Hispanic American	37.6 (9.6)		17.6 (7.8)		0.90 (0.74)	
Education						
<High school	36.2 (10.3)	≤0.0001	19.4 (8.3)	≤0.0001	0.97 (0.77)	NS
High school–associate degree	39.4 (10.0)		16.3 (8.3)		0.86 (0.94)	
Bachelor degree and higher	41.4 (10.1)		14.8 (8.0)		0.80 (0.68)	
CVD risk factor						
BMI, kg/m^2^	0.15	≤0.0001	0.06	NS	0.04	NS
Systolic blood pressure, mm Hg	−0.13	0.0001	0.02	NS	0.06	NS
Diastolic blood pressure, mm Hg	−0.07	≤0.05	0.05	NS	0.06	NS
ADA diabetes category						
Normal glucose	40.3 (10.1)	NS	15.5 (8.1)	NS	0.82 (0.87)	NS
Diabetes	39.8 (10.2)		16.6 (8.6)		0.87 (0.67)	
Smoking						
Never smoker	39.6 (9.9)	NS	15.8 (7.7)	NS	0.82 (0.95)	NS
Former smoker	39.8 (10.4)		16.1 (8.9)		0.88 (0.70)	
Current smoker	41.9 (10.1)		16.3 (8.1)		0.77 (0.73)	
Lipids						
Total‐cholesterol, mg/dL	0.04	NS	−0.03	NS	−0.03	NS
HDL‐cholesterol, mg/dL	0.03	NS	−0.08	≤0.05	0.02	NS
Triglycerides, mg/dL	−0.03	NS	0.05	NS	0.02	NS
Inflammation						
C‐reactive protein, mg/L	0.03	NS	0.08	≤0.05	0.03	NS
Interleukin‐6, pg/mL	−0.03	NS	0.10	≤0.005	0.08	≤0.05
sIL‐2rα	−0.04	NS	−0.002	NS	−0.03	NS
sICAM‐1	−0.06	NS	0.13	≤0.005	0.10	≤0.05
Seasonality						
Spring	39.8 (10.5)	NS	16.4 (8.0)	NS	0.79 (0.57)	≤0.005
Summer	38.8 (9.4)		17.5 (8.2)		1.17 (0.72)	
Fall	39.9 (9.6)		15.8 (8.5)		0.94 (0.76)	
Winter	41.3 (10.3)		15.6 (8.3)		0.78 (0.95)	
Serology						
*Chlamydophila pneumoniae* antibodies						
0	40.1 (10.1)	NS	15.5 (8.6)	NS	0.77 (0.57)	NS
1+	40.7 (9.4)		16.0 (8.3)		0.82 (0.71)	
2+	39.1 (10.5)		16.5 (7.9)		0.91 (0.99)	
3 to 4+	41.3 (10.1)		15.2 (8.6)		0.81 (0.76)	
CMV antibodies, EU/mL	−0.20	≤0.0001	0.41	≤0.0001	0.11	≤0.005
HSV antibodies, EU/mL	−0.15	≤0.0001	0.10	≤0.01	0.07	NS
Hepatitis A antibodies (index value)	0.22	≤0.0001	−0.14	≤0.0001	−0.05	NS
*Helicobacter pylori* antibodies (index value)	−0.11	≤0.005	0.11	≤0.005	0.07	NS
Lymphocytes						
Lymphocyte count	0.08	≤0.05	0.10	≤0.005	−0.003	NS
%CD4^+^ cells	NA	NA	−0.26	≤0.0001	−0.16	≤0.0001
%Th1 cells	—	—	NA	NA	0.45	≤0.0001

Data are means (SD) or Pearson correlation coefficients. CVD indicates cardiovascular disease; BMI, body mass index; HDL, high‐density lipoprotein; sIL‐2rα, soluble interleukin‐2 receptor α; sICAM‐1, soluble intercellular adhesion molecule‐1; CMV, cytomegalovirus; HSV, herpes simplex virus; EU, ELISA unit; NS, nonsignificant; NA, not available.

**Figure 4.**
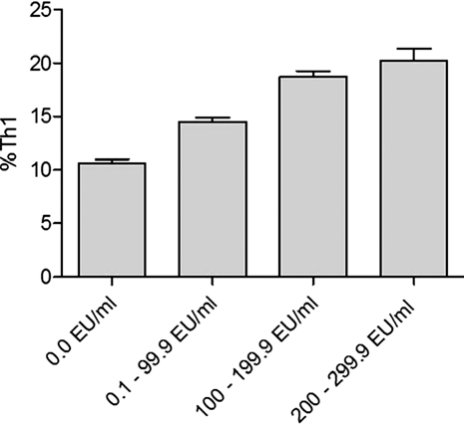
Distribution of %Th1 cells by category of CMV antibody titer. Mean (SEM) values of %Th1 cells are shown by category of CMV antibody titer. CMV categories were defined as 0.0 ELISA units (EU)/mL, 0.1 to 99.9 EU/mL, 100 to 199.9 EU/mL, and 200 to 299.9 EU/mL. CMV indicates cytomegalovirus; SEM, standard error of the mean.

We observed significant differences in all 3 cell phenotypes by serologies related to common infections (Table 3). Higher %CD4 was associated with hepatitis A seropositivity. Notably, higher %Th1 was strongly associated with CMV seropositivity, with ≈17% shared variance between anti‐CMV antibody titer and %Th1. Figure 4 shows the distribution of %Th1 cells by category of CMV antibody titer.

Among the cell phenotypes, %Th1 and %Th2 were both negatively associated with %CD4 cells. Correlation of %Th1 and %Th2 showed ≈20% shared variance (unadjusted *r*^2^=0.20). No cell phenotype was associated with sIL‐2rα, a purported marker of T‐cell activation,^35^ but we did observe associations with the soluble form of ICAM‐1, a major endothelial adhesion molecule needed for T‐cell trafficking (Table 3).

Potential independent variables were included in a backward elimination regression to develop models for %CD4, %Th1, and %Th2 (Table 4). For %CD4, the main independent variables were race/ethnicity, season, and CMV titer, with relatively small contributions by sex and IL‐6 level; the final model *R*^2^ was 0.15, indicating the model could account for 15% of the %CD4 variance. For %Th1, the association with CMV titer was relatively large, with relatively small contributions from age, sex, seasonality, and IL‐6; the final model accounted for 21% of the %Th1 variance. In the final model for %Th2, the main contributions came from race and season; the model accounted for 6% of the %Th2 variance.

**Table 4.** Final Regression Models for %CD4, %Th1, and %Th2

Variable (Increment)	%CD4		%Th1		%Th2	
	Coefficient	*P* Value	Coefficient	*P* Value	Coefficient	*P* Value
Age (10‐year increase)	**—**	NS	−0.86	≤0.005	—	NS
Sex (male vs female)	−3.17	≤0.005	1.12	≤0.05	—	NS
Race (vs white)						
Asian American	−7.73	≤0.001	0.09	NS	0.31	≤0.01
African American	−9.01	≤0.001	1.08	NS	0.33	≤0.001
Hispanic American	−4.34	≤0.001	0.78	NS	0.10	NS
Season (vs winter)						
Spring	0.16	NS	1.44	≤0.05	0.14	NS
Summer	−7.39	≤0.001	3.03	≤0.01	0.53	≤0.001
Fall	−0.23	NS	0.35	NS	0.25	≤0.001
CMV (vs 0.0 EU/mL)						
0.1 to 99.9 EU/mL	−4.84	≤0.001	4.02	≤0.001	**—**	NS
100 to 199.9 EU/mL	−4.48	≤0.005	7.94	≤0.001	**—**	NS
200 to 299.9 EU/mL	−7.35	≤0.001	9.30	≤0.001	—	NS
IL‐6, pg/mL	−1.19	≤0.01	0.50	≤0.05	**—**	NS
Model *R*^2^	0.15	—	0.21	—	0.06	—

Backward elimination regression was used to develop multivariable models for T‐cell indices. After forcing age, sex, and race/ethnicity into the models, BMI, season, CMV antibody titers, *Helicobacter pylori* index values, hepatitis A index values, HSV index values, *Chlamydophila pneumoniae* arbitrary fluorescent units, CRP, WBC, IL‐6, smoking status (never, former, current), total cholesterol, HDL‐cholesterol, systolic blood pressure, diastolic blood pressure, use of hypertension medication, diabetes status, and education level were entered as candidate starting variables. CMV indicates cytomegalovirus; IL, interleukin; EU, ELISA unit; NS, nonsignificant; BMI, body mass index; CRP, C‐reactive protein; WBC, white blood cell; HDL, high‐density lipoprotein.

### Association of %CD4, %Th1, and %Th2 Cell Phenotypes With Atherosclerosis

Table 5 describes multivariable adjusted models in which the association of traditional CVD risk factors, biomarkers of inflammation, and %CD4, %Th1, and %Th2 were evaluated with measures of subclinical disease. There was no association of the T‐cell phenotypes with the presence of CAC (ie, CAC=0 versus CAC>0; results not shown). However, the amount of CAC (lnCAC in those with nonzero CAC) was significantly associated with %Th1 after adjustment for age, male sex, and former and current smoking status (the significant variables from the backward selection). For each 10% unit increase in the %Th1 variable, lnCAC increased by 0.25 arbitrary units (AU).

**Table 5.** Final Regression Models for Atherosclerosis with %CD4, %Th1, and %Th2

Variable (Increment)	lnCAC (CAC>0) (n=552)		cIMT‐common (n=905)	
	Coefficient	*P* Value	Coefficient	*P* Value
Age (10‐year increase)	0.71	≤0.001	0.08	≤0.001
Sex (male vs female)	0.81	≤0.001	0.06	≤0.001
Race (vs white)				
Asian American	−0.36	NS	−0.01	NS
African American	−0.36	NS	0.03	NS
Hispanic American	−0.38	NS	−0.03	≤0.05
Blood pressure				
Systolic BP (10 mm Hg)	**—**	NS	0.02	≤0.001
Diastolic BP (10 mm Hg)	**—**	NS	−0.02	≤0.05
BMI (5 kg/m^2^)	**—**	NS	0.01	≤0.01
Smoking (vs never smoker)				
Former	0.39	≤0.05	**—**	NS
Current	0.61	≤ 0.05	**—**	NS
Cell‐based analytes				
%Th1 (1 SD, 8%)	0.20	≤0.01	0.01	≤0.01
%Th2 (1 SD, 0.8%)	**—**	NS	−0.01	≤0.01
Model *R*^2^	0.17	—	0.33	—

Robust regression was used to develop multivariable models for subclinical disease as described in Methods and Results. The models started with all 3 cell‐based indices, some of which may have been dropped if nonsignificant. CAC indicates coronary artery calcification; cIMT, carotid intimal media thickness; BP, blood pressure; BMI, body mass index.

For the CC IMT, %Th1 and %Th2 were both significant variables (positive and negative associations, respectively) after adjustment for age, male sex, race/ethnicity, blood pressure, and BMI. For each 10% unit increase in %Th1, CC IMT increased by 0.02 mm, whereas for each 1% increase in %Th2, CC IMT decreased by 0.02 mm. There was no statistically significant association of the T‐cell phenotypes with the internal carotid IMT. There was no evidence of an interaction with seasonality in either the lnCAC or CC IMT model.

## Discussion

The major findings of this population‐based multiethnic study of Th1 and Th2 cells and measures of subclinical atherosclerosis are: (1) T‐helper bias toward Th1 cells is associated with 2 different estimates of subclinical atherosclerosis in a multiethnic healthy population; and (2) a measure of CMV infectious burden is strongly associated with Th1 bias.

### Association of T‐Helper Bias With Atherosclerosis

Our findings of associations of Th1 bias with atherosclerosis are consistent with the substantial evidence in the literature that Th1 cells are important in the initiation and progression of atherosclerosis. In human clinical studies, activated CD4^+^ T lymphocytes have been identified in atherosclerotic lesions,^36^ with evidence that these cells are predominately T‐helper type 1 cells.^13–14,13–38^ In murine models, Th1 bias has a profound effect on early atherosclerosis,^6^ and Th1 cells are a main source of IFN‐γ, which has been demonstrated to contribute to murine atherosclerosis^7–9,39^ through multiple mechanisms, including the activation of macrophages and endothelial and smooth muscle cells, the inhibition of macrophage cholesterol efflux, and weakening the fibrous cap.^40^ However, it is important to note that a cross‐sectional study like ours cannot establish causality, and prospective studies are needed.

The role of Th2 cells in atherosclerosis is less certain than that for Th1 cells. In some murine models the key Th2 cytokine IL‐4 has antiatherogenic properties,^6^ although there have been inconsistent results.^20–21,41^ We observed a significant negative association of Th2 bias with common carotid IMT, consistent with an antiatherogenic effect. However, human diseases in which Th2 cells have been implicated such as systemic lupus erythematosis and asthma are not atheroprotective and in fact are associated with increased atherosclerotic burden.^42–43^ Our current hypothesis is that such diseases increase atherosclerotic burden because of markedly increased inflammation (ie, innate immunity) despite the bias toward Th2 helper function.

### Association of T‐Helper Bias With Infectious Burden

Although it has been established that acute CMV infection invokes a predominant Th1 bias in humans,^44^ the observations of strong associations in apparently healthy people between CMV serology, including those with latent infection, and CD4 and Th1 lymphocyte subpopulations are novel and suggest a possible role of chronic CMV infection in atherosclerosis.

Previous results regarding CMV infection and atherosclerosis have been inconsistent, especially with regard to the presence of CMV in human plaques.^45^ In MESA, no associations were found between baseline measures of atherosclerosis and individual pathogens, including CMV antibody titer, or with overall pathogen burden in a subset of participants.^46^

Possible explanations for these discrepancies include the aforementioned possibility of reverse causality; a relatively small sample size for population studies, and CVD measures that do not evaluate the spectrum of disease severity.

Alternatively, and in our view more likely, because CMV antibody levels were found to be associated with inflammatory biomarkers in MESA,^26^ CMV infection may only have an indirect role in the development of atherosclerosis by promoting chronic immune activation (and Th1 biasing) and inflammation (eg, increased circulating IL‐6 levels), responses that may vary depending on other underlying factors. For example, human immunodeficiency virus (HIV) infection has been reported to enhance the immune response against CMV, and CMV‐specific T cell responses have been independently associated with IMT in HIV‐infected patients.^47^

### Population Distribution and Physiological Correlates of T‐Helper Cells

To our knowledge, this is the first large‐scale epidemiologic study of T‐helper cell bias in a healthy, multiethnic population. Although smaller cellular immunology studies have been relatively common in specific clinical settings,^10–11,14,36,48^ performing such investigations on a larger scale in the context of a multicenter epidemiological study poses new challenges.^49–51^

The finding that healthy people vary consistently from each other with respect to T‐helper bias, without the influence of overt diseases, strongly suggests that such bias precedes clinical disease and is consistent with the view that T‐helper bias may influence disease occurrence. The observation that a significantly larger fraction of lymphocytes was CD4^+^ cells in whites compared with the other ethnicities was unexpected and may be a result of unknown differences in infectious agents, other than those studied in MESA, such as helminthic parasites and protozoa, which evoke T‐helper cell responses. Alternatively, race/ethnicity‐specific genetic variation may play a role, which is currently being explored in genome‐wide association studies using data from the NHLBI MESA‐SHARe project (http://www.ncbi.nlm.nih.gov/projects/gap/cgi-bin/study.cgi?study_id=phs000420.v3.p2).

The findings that the fraction of CD4^+^ lymphocytes was lower in winter, whereas the fractions of the CD4^+^ cells that were Th1 and Th2 cells were higher, are consistent with the general thinking regarding increased infectious burden and/or allergic burden in the different seasons.^34^ The former result is consistent with recent small studies in healthy volunteers with allergic hay fever.^52^ The latter findings, to our knowledge, have not been observed before.

### Limitations and Future Studies

We note several limitations of the present study. The data are cross‐sectional in nature. The sample size, although the largest assembled for this type of cellular epidemiology work, is too small—and the follow‐up period following MESA exam 4 too short—for meaningful coronary heart disease incident events to accumulate. To address this, we are currently working on both plasma‐based estimates and flow‐cytometric analyses of cryopreserved cells,^51^ to determine Th bias, either of which would allow us to exploit nested case–control methodologies. Although we have measured T‐cell subsets in peripheral blood, not within lymph nodes, a recent report suggests that it is unlikely we have missed important features of the immune system.^53^

Importantly, we have only measured Th bias at 1 point in the MESA participants (during exam 4). As we^31^ and others^54^ have shown, regression dilution resulting from analytical and within‐subject variances reduces the apparent strength of most associations, so it is reasonable to assume the actual association of Th bias with atherosclerosis is somewhat greater than we have observed. Because several of the variables were measured earlier than the cell indices (eg, IL‐6, CRP, and the antibody levels were measured in exam 1), some associations may be greater than we have observed. Finally, at the time we developed the protocol for MESA‐Inflammation, readily available methods did not yet exist for estimating several other T‐cell subsets, such as Th17 cells and Foxp3^+^ regulatory T cells; we hope to include these cells in future studies.

## Conclusions

In conclusion, we have demonstrated that CMV is strongly associated with Th1 cell bias and that Th1 bias is associated with subclinical atherosclerosis, independent of known CVD risk factors, in a multiethnic population. These findings are consistent with the role of Th1 cells in atherosclerosis and may provide a candidate mechanism governing the relationships of CMV with cardiovascular disease. They also suggest the importance of prospective studies of T‐helper cell biasing in CVD.
